# P207: A cluster of panton- and valentin- producing Staphylococcus aureus infection at a departmental hospital in Benin: possible association with consumption of contaminated food

**DOI:** 10.1186/2047-2994-2-S1-P207

**Published:** 2013-06-20

**Authors:** TA Ahoyo, C Le Brun, M Makoutode, S Baba-Moussa, Y Piemont², K Dramane, G Prevost², A Sanni

**Affiliations:** 1 Laboratory of Biochemistry and Molecular Biology, Faculty of Sciences and Techniques, University of Abomey, Abomey, Benin; 2 Institute of Bacteriology, University Hospital, University Louis Pasteur, UPRES EA-3432, Strasbourg, France; 3 IRSP, University of Abomey, Abomey, Benin

## Introduction

A three-month period in 2005, two distinct types of methicillin-sensitive *Staphylococcus aureus* (one producing of Panton-Valentine leukocidin (PVL) and the other not) were isolated from bronchial specimens of paediatric inpatient unit at the Zou/Collines Departmental Hospital (CHDZ/C), who had been previously cared for by the nurses. The source of outbreak was probably a faulty contamination of specific food consumed by patients.

## Objectives

we aim to determine the source of particular *S. aureus* strains and possible relationships with hospital environment.

## Methods

An investigation was conducted that involved screening of all inpatients receiving a specific food, hospital environment sampling and the follow-up of cases until the end of hospital stay. Isolates were identified, tested for antimicrobial susceptibility and analysed for PVL,LukE/LukD, and enterotoxin A production. Pulse Field Gel Electrophoresis (PFGE) was performed to establish the clonality of the strains.

## Results

A total of 36 infected inpatients with *S. aureus* were identified. Twenty-eight cases of pneumonia were discovered and PVL-producing *S. aureus* concerned 61%. By PFGE an indistinguishable PVL-producing *S. aureus* was identified in the food served, 28 patients, the keyboard and faucet handles in their respective room. Enhanced hygiene measures, particular hand hygiene, terminated the outbreak.

## Conclusion

Our finding suggest an associated between environmental contamination and patient infection, not limited to the patients’ rooms*.* Transmission of PVL-producing *S. aureus* can be prevented in the hospital by a combination of decontamination of the environment, and the promotion of hand hygiene.

## Disclosure of interest

None declared

